# Application of an improved naive Bayesian analysis for the identification of air leaks in boreholes in coal mines

**DOI:** 10.1038/s41598-022-20504-0

**Published:** 2022-09-27

**Authors:** Hong-yu Pan, Sui-nan He, Tian-jun Zhang, Shuang Song, Kang Wang

**Affiliations:** College of Safety Science and Engineering, Xi’an University of Science and Technology, Xi’an, 710054 People’s Republic of China

**Keywords:** Coal, Environmental sciences, Engineering

## Abstract

Borehole extraction is the basic method used for control of gases in coal mines. The quality of borehole sealing determines the effectiveness of gas extraction, and many influential factors result in different types of borehole leaks. To accurately identify the types of leaks from boreholes, characteristic parameters, such as gas concentration, flow rate and negative pressure, were selected, and new indexes were established to identify leaks. A model based on an improved naive Bayes framework was constructed for the first time in this study, and it was applied to analyse and identify boreholes in the 229 working face of the Xiashijie Coal Mine. Eight features related to single hole sealing sections were taken as parameters, and 144 training samples from 18 groups of real-time monitoring time series data and 96 test samples from 12 groups were selected to verify the accuracy and speed of the model. The results showed that the model eliminated strong correlations between the original characteristic parameters, and it successfully identified the leakage conditions and categories of 12 boreholes. The identification rate of the new model was 98.9%, and its response time was 0.0020 s. Compared with the single naive Bayes algorithm model, the identification rate was 31.8% better, and performance was 55% faster. The model developed in this study fills a gap in the use of algorithms to identify types of leaks in boreholes, provides a theoretical basis and accurate guidance for the evaluation of the quality of the sealing of boreholes and borehole repairs, and supports the improved use of boreholes to extract gases from coal mines.

## Introduction

One of the most common and dangerous natural risks associated with coal mining is methane, which can mix with air and cause disasters. Extraction of gases from coal mines is a fundamental measure taken to prevent and control disasters and accidents^1,2^. Drainage boreholes are used to extract gas from coal seams^3^. However, the concentration of gas extracted from coal seams by boreholes in China is generally low because of leaks. Air flows through a channel into a borehole and reduces the negative pressure to enable gas extraction. As a result, low concentrations of gas are extracted by boreholes^4,5^. The effective identification of the presence and types of gas leaks is vital to improve the efficiency of gas extraction.

Studies of the mechanism of borehole leakage have led to the development of physical models. Zhang T^6^ explained that air leakage was caused by a local change in the strain^7,8^ around a borehole. Zhang C^9^ studied the mechanism of air leakage in the cracks around a borehole and concluded that the leakage mechanisms of fractures around boreholes differed depending on the extraction stage. This insight provided a theoretical basis for the classification and identification of leaks in boreholes. To further analyse the flow state and characteristic changes of air leaks in boreholes, some scholars constructed a physical model to determine the mechanism of leakage. Zhang J^10,11^ combined numerical simulations of the leakage mechanism around a borehole in coal with the rheological and viscoelastic–plastic characteristics of coal to build a dynamic leakage model of the borehole. Based on an analysis of flow coupling between methane and air in borehole fractures, Fan J^12^ constructed a flow model of air leakage coupling components in boreholes by using the finite difference method (FDM). Zhang Y^13^ constructed a physical model of air leakage in boreholes and classified three types of leaks according to their source, i.e., roadway fissure zones, borehole fissure zones, and materials used in sealing sections of boreholes. Wang Z^14^ analysed the mechanism of air leakage from boreholes by numerical simulation and established a dynamic leakage model of drainage boreholes. Wang H^15^ and Zhang Y^16^ discussed the influence of air leakage on gas concentration by studying the influence of factors around roadways and boreholes and constructed an air-gas mixed-flow coupling model. Their physical model explained the mechanisms of gas extraction and air leakage in boreholes and provided a theoretical basis for the classification of air leaks from boreholes. However, the construction of a physical model of air leakage in a drilling hole is complicated and cannot be applied quickly to guide field practice. Therefore, there is still a need for an efficient mathematical model for identification of leaks.

Advances in computer science, applied mathematics and artificial intelligence have promoted in-depth research on identification models for use in coal mining^17–22^. However, the algorithms used to construct these models are subject to limitations. The hierarchical cluster analysis method cannot redistribute existing data and has a small number of iterations. The chaotic immune particle swarm optimization-probabilistic neural network (CIPSO-PNN) optimizes the PNN, but the process of finding the best solution is long, and the model is complex. In Fisher's discriminant analysis, the number, representativeness and correctness of the learned samples directly impact the recognition accuracy of the model. In addition, the algorithms of existing discrimination classification models cannot adapt to differences in the relationships of various data characteristics with multiparameter nonlinearity, which is important for discrimination of leaks in extraction boreholes. naïve Bayes classification, a classification method based on the Bayes principle and independent assumption of feature conditions, has stable classification efficiency^23^. Compared with the above classification algorithms, decision trees and artificial neural networks perform better on small amounts of sample data and have the minimum error rate, and they have been widely used in coal mines^24,25^. Therefore, they have been applied to identify in air leaks in gas boreholes. However, because of low sensitivity to linear data, improvements are needed.

In summary, research is now relatively mature for the development of models for leaks in boreholes for gas extraction based on studies of the mechanism of air leakage, and models are widely used in the field of coal mining. However, research is limited on using machine learning methods to analyse multisource characteristic information about air leakage and establish a mathematical model for the recognition of leaks from boreholes. In this study, we collected data for leaks from boreholes and applied multisource data fusion theory (MDF) and principal component analysis (PCA). We also improved the traditional naive Bayesian classification (NBC) system and established mathematical models to identify types of air leaks from boreholes. In this study, this model fills a gap by supporting an algorithm to identify types of leaks in boreholes used to extract gases from coal mines, provides a theoretical basis and accurate guidance for the evaluation of the quality of the sealing of boreholes and borehole repairs, and supports the improvement of the application of boreholes to extract gases from coal mines.

## Construction of an improved naive Bayesian model for the identification of air leaks from gas drainage boreholes

### Feature information selection

According to previous studies^4,15,26–31^, air leaks from gas drainage boreholes can be divided into the three types shown in Table 1.

**Table 1:** Types of borehole leaks.

	Type	Distance	Reason
I	Air leakage from the orifice of the gas drainage borehole	0–2 m	The pipe wall broke and leaks developed
II	Air leakage from the mid-end seal section of the gas drainage borehole	2–9 m	Cracks developed in the seal
III	Air leakage from the deep coal of the gas drainage borehole	9–12 m	The strength of the seal was insufficient

**Figure 1:**
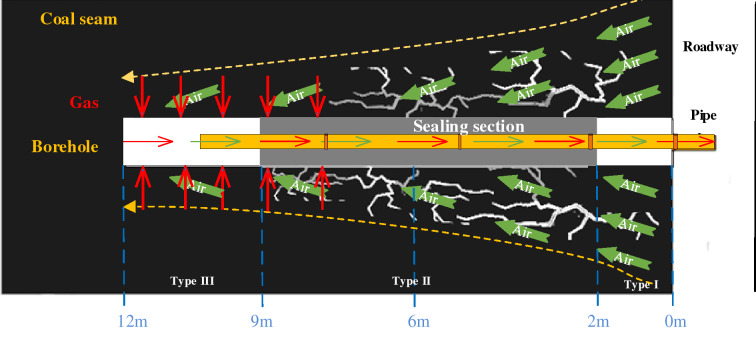
Air leakage characteristics of a borehole for extraction of gas.

In Fig. 1, there are many cracks in the coal seam. Due to the poor sealing effect, air from the roadway enters the borehole through cracks in the coal seam, which leads to the leakage of the borehole. In addition, the connections between the extraction pipes are not close, which results in low extraction concentrations. In this paper, according to the actual situation of the gas drainage borehole in the 229 working face of the Xiashijie Coal Mine, eight characteristics can reflect the gas drainage effect of the borehole, including A_1_: extraction flow, A_2_: gas concentration at 0 m, A_3_: gas concentration at 2 m, A_4_: gas concentration at 6 m, A_5_: gas concentration at 9 m, A_6_: gas concentration at 12 m, A_7_: negative pressure at the orifice and A_8_: negative pressure at the extraction, and they are used in the model for the identification of leaks in boreholes for gas extraction.

### Identification model construction

**Figure 2:**
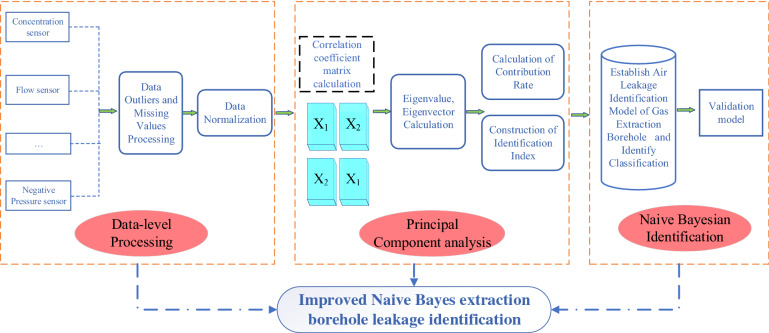
Flow chart of building the model.

A naive Bayes classifier (NBC) and the eight characteristics (above) were used as the main theory for model construction. Since the NBC could not accommodate the missing data for air leakage in gas extraction boreholes, and since the identification and classification accuracy of information with strong correlations is not high, some data easily have a greater impact on the overall model^32^. As shown in Fig. 2, by using MDF and principal component analysis (PCA) to improve the traditional NBC, a model for the identification of air leaks from a borehole for gas extraction was established as follows:

#### Data preprocessing

The existing **m**-dimensional sample data of gas drainage borehole leakage ${\mathbf{x}} = \left( {x_{1} ,x_{2} \ldots x_{m} } \right)$ with n independent observations, $\left( {{\mathbf{x}}_{{\mathbf{1}}} {\mathbf{,x}}_{{\mathbf{1}}} {\mathbf{ \ldots x}}_{{\mathbf{n}}}^{{\text{T}}} } \right)$, is used as the observation sample to build the gas drainage borehole leakage data matrix:

1 $${\mathbf{X}} = \left[ {{\mathbf{x}}_{{\mathbf{1}}} {\mathbf{,x}}_{{\mathbf{2}}} {\mathbf{ \ldots x}}_{{\mathbf{n}}} } \right]^{{\mathbf{T}}} = \left[ {\begin{array}{*{20}c} {x_{11} } & {x_{12} } & \ldots & {x_{1m} } \\ {x_{21} } & \ldots & \ldots & \ldots \\ \ldots & \ldots & {x_{ij} } & \ldots \\ {x_{n1} } & \ldots & \ldots & {x_{nm} } \\ \end{array} } \right]$$

${\mathbf{x}}_{{\mathbf{i}}} = \left( {x_{i1} ,x_{i2} \ldots x_{im} } \right)$ represents the observation sample of group *i*, *i* = 1,2…,n, and$x_{ij}$ represents the jth variable of the ith group of observation samples, where *j* = 1,2…,m.

Following MDF theory, the training samples for gas drainage borehole leaks are processed at the data level^33^, and the processed data are standardized with Eqs. (2)–(4).

2 $$x_{ij}^{1} = \frac{{x_{ij} - \overline{x}_{j} }}{{\sqrt {s_{jj} } }}\quad i = 1,2....{\text{n}};\;\;j = 1,2....{\text{m}}$$

3 $$\overline{x}_{j} = \frac{1}{n}\sum\limits_{i = 1}^{n} {x_{ij} }$$

4 $$s_{ij} = \frac{1}{m - 1}\sum\limits_{j = 1}^{m} {\left( {x_{ij} - \overline{x}_{j} } \right)^{2} }$$

In Eqs. (2)–(4), $x_{ij}^{1}$ represents the standardized single sample data, $\overline{x}_{j}$ represents the sample mean for the same characteristic information, and $s_{jj}$ represents the covariance of single sample data. Equations (2)–(4) can eliminate the influence of the data dimension. The standardized gas drainage borehole leakage data are still expressed in X.

#### Principal component selection

The correlation coefficient matrix of the gas drainage borehole leakage sample data after standardization is calculated as follows:

5 $$R = \left[ {r_{ij} } \right]_{n*n} = \frac{1}{m}XX^{{\mathbf{T}}}$$

where

6 $$r_{ij} = \frac{1}{m - 1}\sum\limits_{i = 1}^{m} {x_{il} x_{lj} \quad i,j = 1,2....{\text{n}}}$$

The characteristic equation of the sample correlation matrix **R** is obtained with **k** eigenvalues and the corresponding **k** unit eigenvectors:

7 $$\begin{gathered} \left| {{\mathbf{R}} - \lambda {\mathbf{I}}} \right| = 0 \hfill \\ \lambda_{1} \ge \lambda_{2} \ge \lambda_{3} \ge \ldots \lambda_{m} \hfill \\ \end{gathered}$$

In Eq. (7), $\lambda$ is the characteristic value of the characteristic equation corresponding to the characteristic information, and the values are sorted according to the size of the characteristic value, from large to small.

The cumulative contribution rate and cumulative variance contribution rate are calculated as follows:

8 $$z = \frac{{\lambda_{k} }}{{\sum\limits_{i}^{m} {\lambda_{m} } }}$$

9 $$z_{i} = \sum\limits_{j = 1}^{k} {\left( {\frac{{\lambda_{k} }}{{\sum\limits_{i}^{m} {\lambda_{m} } }}} \right)}$$

The principal component $z_{i} \ge 85\%$ is determined to reduce the dimensionality and eliminate information overlap.

#### Construction of new indexes

The unit eigenvector corresponding to the first *k* principal components is obtained:

10 $$a_{i} = \left( {a_{1i} ,a_{2i} \ldots a_{ni} } \right)^{{\mathbf{T}}} ,\;\;i = 1,2,3 \ldots {\text{k}}$$

Linear transformation with *k* unit eigenvectors as coefficients yields:

11 $$Y_{i} = a_{i}^{T} {\mathbf{x}}\quad i = 1,2,3 \ldots {\text{k}}$$

That is, after orthogonal transformation, potentially correlated variables or influencing factors in the gas drainage borehole leakage data are linearly combined to obtain a set of new linear irrelevant variables, simplify the data structure, extract the data characteristics, and construct a new improved naive Bayes identification index.

**Figure 3:**
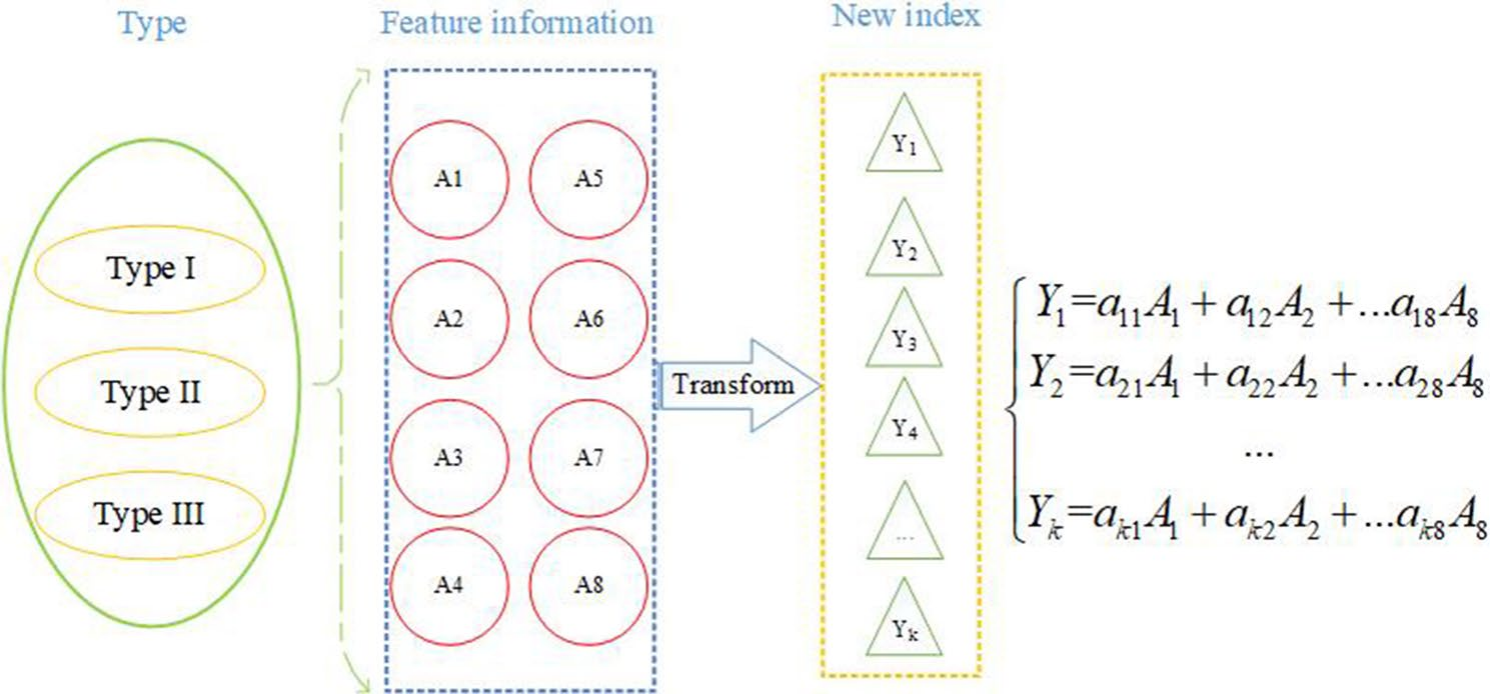
Construction of the new indexes for air leakage from a gas drainage borehole.

As shown in Fig. 3, for the characteristic information obtained in the lower section, the original eight-dimensional sample characteristic information (A_1_, A_2_…, A_8_) is converted into a new p-dimensional identification index (Y_1_, Y_2_…, Y_k_) *k* < 8. The associated characteristic information is combined and retains most of the information of the original variables^34^ while eliminating overlapping information.

#### Modelling

The data matrix ${\mathbf{Y}} = [{\mathbf{y}}_{1} ,{\mathbf{y}}_{2} ...{\mathbf{y}}_{k} ]$ is constructed according to the new leakage index of the drainage borehole. Among them,$y_{i} = [y_{1} ,y_{2} ...y_{n} ]^{{\mathbf{T}}}$, $y_{i}^{\left( j \right)}$ is the jth feature of sample *i*, $y_{t}^{\left( j \right)} \in \{ a_{j1} ,a_{j2} , \ldots a_{jsn} \}$, and $a_{jl}$ is the possible value of the jth feature, *i* = 1, 2, 3… n; *j* = 1, 2, 3… k; *l* = 1, 2, 3… s_n_. The sample category is $G = \{ g_{1} ,g_{2} \ldots g_{T} \}$, $y_{i} \in \{ g_{1} ,g_{2} \ldots g_{T} \}$.

The prior probability and conditional probability of the air leakage category of the extraction borehole are calculated. Because the characteristic information data optimized by the principal component are normally distributed, the Gaussian function is used to determine the conditional probability, as shown in Eqs. (12)–(13).

12 $$P\left( {Y = {\text{g}}_{t} } \right) = \frac{{\sum\limits_{i = 1}^{n} {I\left( {{\text{y}}_{i} = {\text{g}}_{t} } \right)} }}{n}\quad {\text{t = 1,2,3}} \ldots {\text{T}}$$

13 $$P\left( {{\text{y}}_{i}^{(j)} = {\text{a}}_{{jl_{jl} }} \left| {Y = g_{t} } \right.} \right) = \frac{1}{{\sqrt {2\sigma_{{Y = g_{t} }}^{2} } }}e^{{\frac{{ - \left( {q_{{y^{(j)} = a_{jl} }} - u_{{y = g_{t} }} } \right)}}{{2\sigma_{{Y = g_{t} }}^{2} }}}}$$

In Eq. (13), $u_{{y = g_{t} }}$ is the normalized expected value of the sample data of category $g_{t}$; $\sigma_{{Y = g_{t} }}^{{}}$ is the normalized variance of the sample data of category $g_{t}$. The posterior probability is calculated for the given leakage sample data $y_{i} = [y_{1} ,y_{2} ...y_{n} ]^{{\mathbf{T}}}$ of the extraction borehole.

14 $$P\left( {Y = g_{t} } \right)\prod\limits_{j = 1}^{k} {P(Y^{(j)} = y^{(j)} \left| {Y = g_{t} } \right.)}$$

The category of an actual case is determined, and the probability model of gas leakage identification of the extraction borehole is built as shown in Eq. (15):

15 $$G_{{y_{i} }} = \arg \mathop {\max }\limits_{{g_{t} }} P(Y = g_{t} )\prod\limits_{j = 1}^{k} {P(Y^{(j)} } = y^{(j)} \left| {Y = g_{t} } \right.)$$

where $G_{{y_{i} }}$ is the maximum posterior probability value of the corresponding category of the leakage of the extraction borehole.

In the actual extraction process, there are gas drainage boreholes with good drainage effects. When the sealing effect is good, the difference in gas concentration at various positions is small. Combined with the air leakage characteristics of the drainage borehole, the gas concentration at different positions in the drainage borehole is defined as $C_{{y_{i} }}^{\left( b \right)}$, *i* = 1, 2… n, for borehole gas concentration positions *b* = 0, 1, 2…,*b*.

16 $$\frac{{C_{{y_{i} }}^{{\left( {b - 1} \right)}} }}{{C_{{y_{i} }}^{\left( b \right)} }} \ge \frac{{C_{{y_{i} }}^{\left( 0 \right)} }}{{C_{{y_{i} }}^{\left( b \right)} }} \ge 90\%$$

The corresponding borehole is a borehole with a good gas drainage effect, and there is no need to evaluate the type of leakage. Incorporating Eq. (15), the gas drainage borehole leakage identification model can be constructed as follows:

17 $$\left\{ {\begin{array}{*{20}l} {\frac{{C_{{y_{i} }}^{{\left( {b - 1} \right)}} }}{{C_{{y_{i} }}^{\left( b \right)} }} > \frac{{C_{{y_{i} }}^{\left( 0 \right)} }}{{C_{{y_{i} }}^{\left( b \right)} }} \ge 90\% } \hfill \\ {G_{{y_{i} }} = \arg \mathop {\max }\limits_{{g_{t} }} P(Y = g_{t} )\prod\limits_{j = 1}^{k} {P(Y^{(j)} } = y^{(j)} \left| {Y = g_{t} } \right.)} \hfill \\ \end{array} } \right.$$

This identification model can realize the identification of leakage and leakage type.

## Model application

### Data acquisition and preprocessing

**Figure 4:**
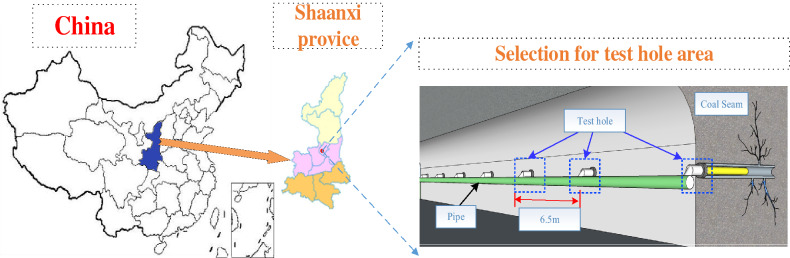
Study area.

The model was applied to the gas drainage borehole of the 229 working face in the Xiashijie Coal Mine of Tongchuan, as shown in Fig. 4. Mainly No. 4 coal is mined in the working face, the thickness of the coal seam is 0 ~ 34.28 m, the original gas content of the coal seam is 3.48 m^3^/t, and the gas pressure is 0.4 MPa, which classifies the mine as a high-gas mine. The gas in the coal seam is extracted by a parallel borehole arrangement.

**Figure 5:**
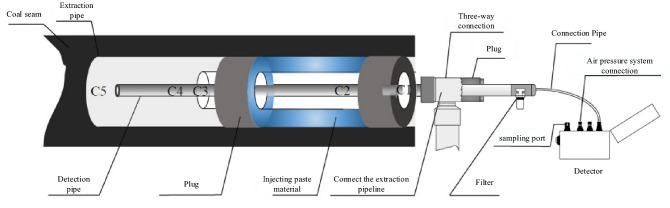
Gas extraction and detection device.

For the purpose of this study, the characteristic information of gas concentration, flow rate and negative pressure at different depths of the borehole were effectively measured. We designed a detection device to connect each borehole and collect data; the device is shown in Fig. 5. By changing the length of the probe, we monitored the gas concentration and extraction flow at different positions in the borehole. The collected data were used to establish the model discussed in this paper.

According to the actual layout of the test boreholes, 30 groups of 240 monitoring data of gas flow, concentration and negative pressure sensors were selected, and they were divided into 18 groups of training samples and 12 groups of test samples according to the ratio of 6:4. The 18 groups of training samples were preprocessed.

Gas drainage borehole leakage data are multidimensional and multivariate^35,36^, with complex correlations. MDF theory was used to preprocess the data^37^. The Newton interpolation method was used to eliminate abnormal values and fill the missing values of the training sample data of the gas leakage borehole^38^ according to Eqs. (18) and (19):

18 $$\begin{aligned} & f\left( {x_{n} ,x_{n - 1} , \ldots ,x_{1} ,x} \right) \\ & \quad = \frac{{f\left[ {x_{n - 1} , \ldots ,x_{1} ,x} \right] - f\left[ {x_{n} ,x_{n - 1} , \ldots ,x_{1} ,x} \right]}}{{x - x_{n} }} \\ \end{aligned}$$

19 $$\begin{aligned} f\left( x \right) & = f\left( {x_{1} } \right) + \left( {x - x_{1} } \right)f\left[ {x_{2} ,x_{1} } \right] \\ & \quad + \left( {x - x_{1} } \right)\left( {x - x_{2} } \right)f\left[ {x_{3} ,x_{2} ,x_{1} } \right] \\ & \quad + \left( {x - x_{1} } \right)\left( {x - x_{2} } \right) \ldots \left( {x - x_{n} } \right)f\left[ {x_{n} ,x_{n - 1} , \ldots ,x_{1} } \right] \\ \end{aligned}$$

The missing value corresponding to the *x-*sequence value was substituted into the calculated value $f\left( x \right)$ to eliminate some abnormal values affecting the overall analysis, fill missing values caused by sensor problems, human operation and other factors, and provide perfect and accurate data for the identification air leaks in boreholes. The complete sample data are shown in Table 2.

**Table 2:** Multisource data table for gas drainage boreholes.

Borehole number	A1: Extraction flow m^3^/min	A2: Gas concentration at 0 m %	A3: Gas concentration at 2 m %	A4: Gas concentration at 6 m %	A5: Gas concentration at 9 m %	A6: Gas concentration at 12 m %	A7: Orifice negative pressure/kPa	A8: Extraction negative pressure/kPa	Type of air leak
1	2.06	5.56	15.40	15.40	15.80	16.42	1.50	20.60	I
2	2.19	5.98	14.56	15.24	15.74	16.08	1.60	20.40	I
3	1.85	6.65	15.24	15.65	15.67	16.22	1.90	21.30	I
4	2.03	5.84	9.84	10.97	11.56	13.25	1.50	21.40	I
5	1.92	2.45	6.85	7.54	7.68	7.93	0.60	20.60	I
6	1.88	6.11	8.66	11.14	13.25	16.40	1.80	20.90	II
7	2.12	7.56	9.25	10.56	16.58	17.65	2.50	22.40	II
8	1.85	4.54	4.56	11.68	11.98	12.38	1.40	19.60	II
9	2.06	6.85	6.84	6.94	15.28	16.34	1.80	20.70	II
10	1.94	5.98	5.84	14.67	15.06	16.57	1.80	21.60	II
11	1.69	8.65	9.64	10.21	10.28	17.68	2.10	19.50	III
12	2.64	7.79	7.81	7.85	7.92	23.21	1.90	20.70	III
13	1.68	8.19	8.21	8.23	8.31	25.80	2.00	20.10	III
14	1.96	6.15	6.15	6.25	6.40	18.21	1.60	19.30	III
15	1.68	5.85	5.88	5.92	8.94	16.01	1.50	21.60	III
16	4.56	10.35	10.56	10.60	10.97	11.35	9.60	21.40	No leakage
17	6.58	19.68	19.89	19.96	20.65	21.14	9.10	20.80	No leakage
18	5.43	13.95	14.00	14.19	14.21	14.37	9.40	21.20	No leakage

Table 2 shows 18 groups of drilling test sample data, of which 15 groups correspond to boreholes with leaks and 3 groups correspond to boreholes without leaks. Because the gas concentrations in boreholes 16, 17, and 18 show little change at 0 m, 2 m, 6 m, 9 m, and 12 m, and the proportion is more than 90% according to model Eq. (16), the drainage effect was good, and there is no need to identify the type of leak. In addition, Table 2 shows that due to the air leakage of boreholes, the concentration decreased greatly from the bottom to the orifice in the boreholes in groups 1–15.

**Figure 6:**
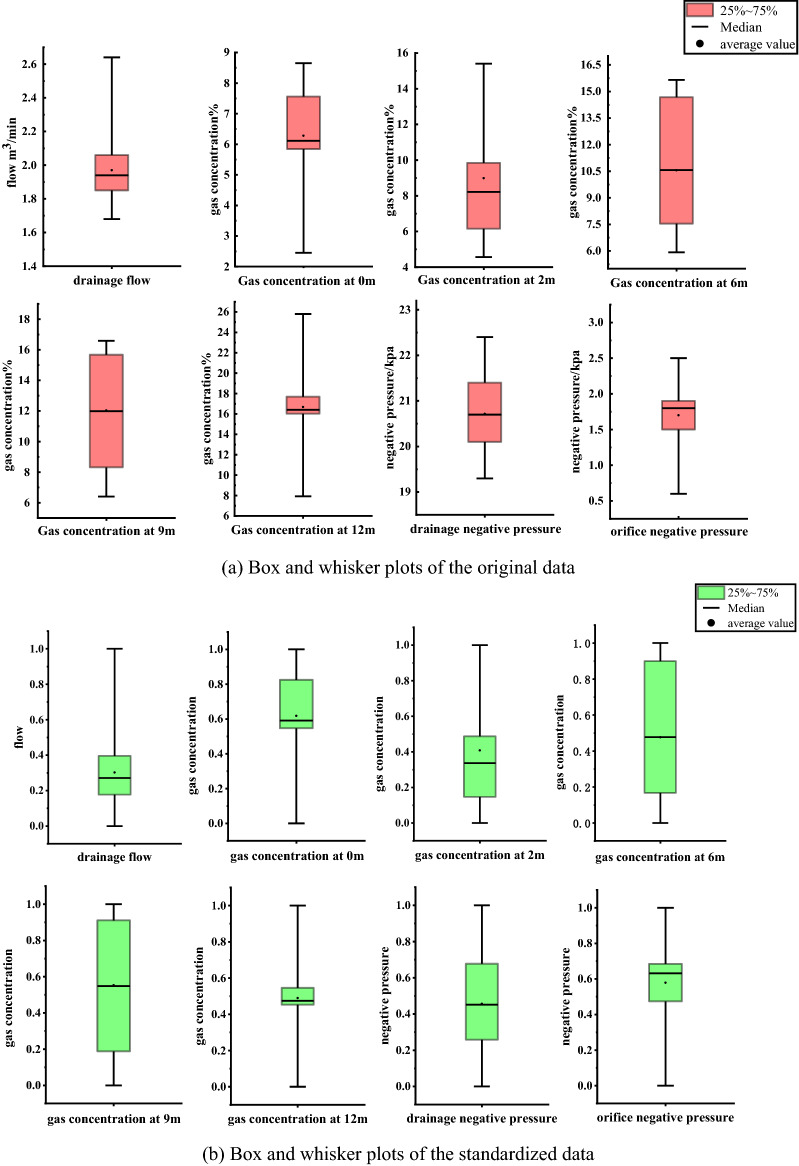
Data comparison.

The leakage data of the first 15 groups of gas extraction boreholes were standardized by Eqs. (2)–(4), as shown in Table 3. The original data were compared with the box diagram of the standardized data. (Box plots can also be used to detect outliers.) Table 2 and Fig. 6a show that the extraction flow rates in the 15 groups of training samples were very similar, approximately 2.0 m^3^/min, which was relatively low. From the negative pressure of extraction to the negative pressure of the orifice, the pressure loss was obvious. Due to the air leakage in the gas drainage borehole, the differences in gas concentrations between samples in each group at 0 m, 2 m, 6 m, 9 m and 12 m were large, and the distribution of gas concentration in the borehole was not uniform. The specific positions of the different types of air leaks in the gas drainage borehole differed. Figure 6b shows that the range and distribution trend of the standardized data were consistent with the original data. After data standardization, the range of the original data was reduced to [0, 1], and the influence of each data dimension was eliminated, thereby optimizing the data for subsequent PCA to determine the new index for the identification of leaks.

**Table 3:** Standardized data for gas extraction boreholes.

Borehole number	A1: Drainage flow	A2: Gas concentration at 0 m	A3: Gas concentration at 2 m	A4: Gas concentration at 6 m	A5: Gas concentration at 9 m	A6: Gas concentration at 12 m	A7: Orifice negative pressure	A8: Extraction negative pressure
1	0.39583	0.50161	1	0.97431	0.92338	0.4751	0.47368	0.41935
2	0.53125	0.56935	0.92251	0.95786	0.91749	0.45607	0.52632	0.35484
3	0.17708	0.67742	0.98524	1	0.91061	0.46391	0.68421	0.64516
4	0.36458	0.54677	0.48708	0.51901	0.50688	0.29771	0.47368	0.67742
5	0.25	0	0.21125	0.1665	0.12574	0	0	0.41935
6	0.20833	0.59032	0.37823	0.53649	0.67289	0.47398	0.63158	0.51613
7	0.45833	0.82419	0.43266	0.47688	1	0.54393	1	1
8	0.17708	0.3371	0	0.59198	0.54813	0.24902	0.42105	0.09677
9	0.39583	0.70968	0.21033	0.10483	0.8723	0.47062	0.63158	0.45161
10	0.27083	0.56935	0.11808	0.89928	0.85069	0.48349	0.63158	0.74194
11	0.01042	1	0.46863	0.4409	0.38114	0.54561	0.78947	0.06452
12	1	0.86129	0.29982	0.19836	0.14931	0.85506	0.68421	0.45161
13	0	0.92581	0.33672	0.23741	0.18762	1	0.73684	0.25806
14	0.29167	0.59677	0.14668	0.03392	0	0.57527	0.52632	0
15	0	0.54839	0.12177	0	0.24951	0.45215	0.47368	0.74194

### New indexes of the model for the identification of leaks

In this study, PCA was used to linearly combine several representative new indexes for identification of leaks. The correlation between the original feature information should be considered to determine whether the PCA is applicable^39,40^. The Kaiser–Meyer–Olkin (KMO) and Butterley sphericity tests were applied in SPSS, as shown in Table 4.

**Table 4:** KMO and Bartlett tests.

**KMO test values for sampling adequacy**	0.664
**Bartlett sphericity test**	
Test value	65.343
Degrees of freedom	28
Significance level	0

As shown in Table 4, the value of Bartlett’s test statistic was 65.343, and the significance level was approximately 0, which was less than the statistical significance level (a = 0.05) specified by SPSS. Thus, the original hypothesis was rejected. That is, the variables in the original data had a statistically significant influence, and the KMO test value was greater than 0.5, which indicated that the air leakage data of the gas drainage borehole were suitable for PCA.

According to the standardized data for air leakage of the gas drainage borehole in Table 3, the correlation coefficient matrix of air leakage characteristics was calculated, as shown in Table 5. The closer the correlation coefficient is to 1, the greater the degree of correlation of the corresponding two groups of characteristics; e.g., the correlation coefficient of gas concentrations at 6 m and 9 m is 0.7447, which indicates a strong correlation. The closer the correlation coefficient is to 0, the smaller the degree of correlation of the corresponding two groups of characteristics; e.g., the correlation coefficient of the gas concentrations at 2 m and 12 m is 0.0593, which indicates a weak correlation. A negative correlation coefficient indicates that the two groups are inversely correlated. For example, the correlation coefficient between the gas concentrations at 9 m and 12 m is − 0.1529.

**Table 5:** Correlation coefficient matrix for air leakage characteristics of gas drainage boreholes.

Index	Extraction flow	Gas concentration at 0 m	Gas concentration at 2 m	Gas concentration at 6 m	Gas concentration at 9 m	Gas concentration at 12 m	Orifice negative pressure	Extraction of negative pressure
Extraction flow	1							
Gas concentration at 0 m	0.0746	1						
Gas concentration at 2 m	0.1594	0.1562	1					
Gas concentration at 6 m	0.0634	− 0.0516	0.6955	1				
Gas concentration at 9 m	0.1202	0.0743	0.5351	0.7447	1			
Gas concentration at 12 m	0.1725	0.8366	0.0593	− 0.1431	− 0.1529	1		
Orifice negative pressure	0.0887	0.9019	0.1599	0.1281	0.3572	0.6947	1	
Extraction of negative pressure	0.1716	0.0028	0.1306	0.1927	0.4870	− 0.0959	0.2453	1

As shown in Table 5, some of the 8 selected gas drainage borehole leakage characteristics are strongly correlated. Using these 8 kinds of characteristic data to identify gas drainage borehole leakage will lead to an incorrect decision, thus affecting the accuracy of the identification model. Therefore, it is necessary to analyse the training sample data via PCA to obtain the eigenvalue and contribution of each feature and select the appropriate principal component to eliminate the strong correlations from the feature data.

The eigenvalues and cumulative contribution rates of the eight types of feature information were obtained through calculations and analysis, as shown in Table 6. The characteristic value of A_1_: extraction flow was the largest, and the contribution of its variance contribution was also the largest. The characteristic value and contributions of A_2_–A_8_ decreased in turn, and the contributions were small; the 6th–8th principal component, A6–A8, were ignored. The cumulative contribution rate of extraction flow and gas concentrations at 0 m, 2 m, 6 m and 9 m reached 95.54%. According to Eq. (9), the contributions of these variables were more than 85%, and they were preliminarily considered as the main identification indexes of the improved naive Bayesian extraction leakage identification model. In a practical sense, the flow rate and concentration are the main variables of gas extraction in the borehole. The concentrations at 0 m, 2 m, 6 m and 9 m can reflect concentration changes in the borehole. The negative pressure has a linear relationship with the concentration and flow rate, and a change in negative pressure affects the concentration and flow rate; thus, it is advisable to select 5 principal components.

**Table 6:** Eigenvalues of correlation coefficients.

Principal component number	Index	Eigenvalue	Variance proportion	Cumulative contribution rate
A1	Extraction flow	2.85632	35.70%	35.70%
A2	Gas concentration at 0 m	2.38875	29.86%	65.56%
A3	Gas concentration at 2 m	1.05432	13.18%	78.74%
A4	Gas concentration at 6 m	0.95071	11.88%	90.63%
A5	Gas concentration at 9 m	0.39309	4.91%	95.54%
A6	Gas concentration at 12 m	0.21658	2.71%	98.25%
A7	Orifice negative pressure	0.10898	1.36%	99.61%
A8	Extraction of negative pressure	0.03125	0.39%	100.00%

**Figure 7:**
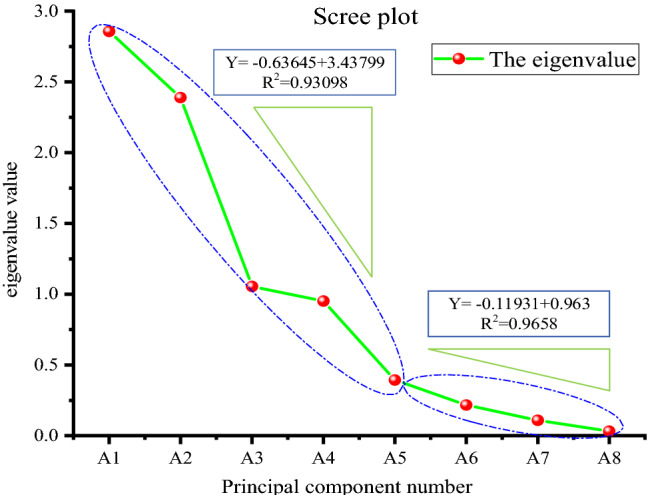
Principal component scree plot.

To further confirm the rationality of this selection, a scree plot was used. A scree plot is a trend map that reflects changes in data characteristics. The steepness of the decrease in eigenvalues shows whether the selected features are correct and reasonable. The scree plot in Fig. 7 shows that the slope k1 of principal components A_1_–A_5_ is − 0.63645, and the trend is steep. The slope k_2_ of principal components A5–A8 is − 0.11931, and the trend is relatively flat. Principal component A5 is an inflection point, and thus, it is reasonable to select these five variables as the principal components.

The original A1–A8 feature information with strong correlations was reconstructed into the selected principal component features Y1–Y5, and the component analysis matrix table of Y1–Y5 was established according to the PCA (Table 7) to establish the new feature information index of gas drainage borehole leakage.

**Table 7:** Component analysis matrix.

Index	Y_1_	Y_2_	Y_3_	Y_4_	Y_5_
A_1_	0.15881	0.03039	0.57660	0.76498	− 0.20849
A_2_	0.44685	− 0.39790	− 0.10068	− 0.06039	0.00492
A_3_	0.34194	0.33001	− 0.34096	0.32301	0.62830
A_4_	0.30134	0.46679	− 0.30251	0.08705	− 0.21162
A_5_	0.37329	0.42654	0.04862	− 0.19592	− 0.43937
A_6_	0.35162	− 0.46037	− 0.06492	0.15732	0.19106
A_7_	0.50706	− 0.25446	0.04217	− 0.23946	− 0.24950
A_8_	0.21746	0.23521	0.66429	− 0.42281	0.47451

Among the new indexes Y_1_–Y_5_, the higher the load coefficient corresponding to the original feature is, the closer the relationship between the feature information and the new indicator, which is the main influence quantity in the new index. According to the component analysis matrix in Table 7, the new index coefficient expression of gas drainage borehole leakage is:

$$\begin{aligned} Y_{1} & = 0.15881A_{1} + 0.44685A_{2} + 0.34194A_{3} + 0.30134A_{4} + 0.37329A_{5} + 0.35162A_{6} + 0.50706A_{7} + 0.21746A_{8} \\ Y_{2} & = 0.03039A_{1} - 0.39790A_{2} + 0.33001A_{3} + 0.46679A_{4} + 0.42654A_{5} - 0.46037A_{6} - 0.25446A_{7} + 0.23521A_{8} \\ Y_{3} & = 0.57660A_{1} - 0.10068A_{2} - 0.34096A_{3} - 0.30251A_{4} + 0.04862A_{5} - 0.06492A_{6} - 0.04217A_{7} + 0.66429A_{8} \\ Y_{4} & = 0.76498A_{1} - 0.06039A_{2} - 0.32301A_{3} - 0.08075A_{4} - 0.19592A_{5} - 0.15732A_{6} - 0.23946A_{7} - 0.42281A_{8} \\ Y_{5} & = - 0.20849A_{1} + 0.00492A_{2} + 0.62830A_{3} - 0.21162A_{4} - 0.43937A_{5} + 0.19106A_{6} - 0.24950A_{7} + 0.47451A_{8} \\ \end{aligned}$$

According to the expression for the characteristic information and the PCA matrix (Table 7), the gas concentrations A_2_–A_6_ in the first principal component Y_1_ at different depths and the load coefficient of negative pressure at orifice A_7_ were greater than those of the other indexes, and this was the main characteristic influence of the first principal component index Y_1_. Therefore, the principal component index of Y_1_ was interpreted as the influencing factor of negative pressure-concentration hole leakage. The load coefficient of A_3_–A_5_ in the second principal component index Y_2_ was higher, so the second principal component index Y_2_ was interpreted as the influencing factor of hole depth-concentration borehole leakage. By analogy, Y_3_ was the influencing factor of negative pressure-flow borehole leakage, Y_4_ is the influencing factor of single flow borehole leakage, and Y_5_ was the negative pressure-hole gas concentration borehole leakage factor.

### Analysis of test sample results

Y_1_, Y_2_, Y_3_, Y_4_ and Y_5_ were used as the new identification indexes of the improved naive Bayesian extraction borehole leakage model, and the prior probability under the new index identification was calculated by Eqs. (12)–(13). According to the numerical relationship between the score of the new index and the leakage type of the above 15 groups of samples, the training was performed by MATLAB platform programming, as shown in Table 8. The final score of each index was taken as the training sample of the improved NBC.

**Table 8:** Improved naive Bayesian training samples.

Borehole number	Y_1_	Y_2_	Y_3_	Y_4_	Y_5_	Type of air leak
1	1.78760	0.75049	− 0.18532	− 0.52607	0.10793	I
2	1.78703	0.67208	− 0.12678	− 0.38738	− 0.00997	I
3	1.93963	0.68025	− 0.19066	− 0.84648	0.19778	I
4	1.29884	0.31448	0.26743	− 0.40568	0.16031	I
5	0.34467	0.30730	0.30644	− 0.09238	0.18911	I
6	1.39628	0.17619	0.08761	− 0.46850	− 0.03418	II
7	1.91326	0.20825	0.52494	− 0.65004	− 0.03102	II
8	0.83946	0.18236	− 0.05390	− 0.14265	− 0.41292	II
9	1.30982	− 0.05108	0.33858	− 0.25551	− 0.20570	II
10	1.57869	0.39450	0.26274	− 0.49350	− 0.25669	II
11	1.37513	− 0.31145	− 0.39515	− 0.44456	− 0.02569	III
12	1.49119	− 0.51862	0.55055	0.35058	0.08350	III
13	1.42849	− 0.65358	− 0.19528	− 0.34884	0.21311	III
14	0.78195	− 0.56312	− 0.01172	0.10143	0.00570	III
15	0.93027	− 0.22577	0.35894	− 0.47733	0.28984	III

**Figure 8:**
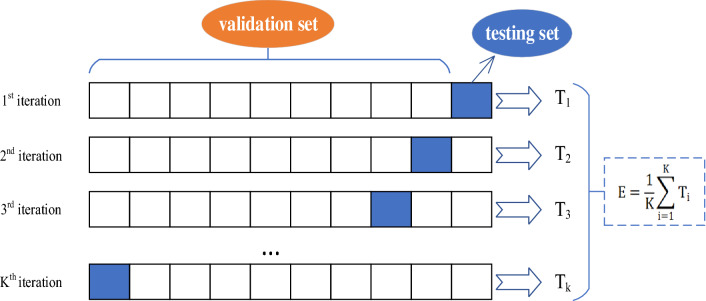
k-fold cross-validation schematic diagram.

To verify the accuracy and reliability of the model, 12 sets of gas drainage borehole data corresponding to three different types of leaks in the Xiashijie Coal Mine were collected as test samples, as shown in Table 9. A total of 240 data points from 30 groups of training samples and test samples were divided into a verification set and a test set according to a ratio of 0.6, which prevented a poor model identification rate and overfitting caused by a verification set that was too large as well as inaccurate model verification caused by a test set sample that was too small. In this study, we adopted the hold-out verification method, namely, the twofold cross-validation method. The schematic diagram of k-fold cross-validation is shown in Fig. 8^41,42^. The data set was divided into a training set and a test set for verification, and the average and accuracy of the final verification results were calculated.

**Table 9:** Test sample data.

Borehole number	A1: Extraction flow	A2: Gas concentration at 0 m	A3: Gas concentration at 2 m	A4: Gas concentration at 6 m	A5: Gas concentration at 9 m	A6: Gas concentration at 12 m	A7: Orifice negative pressure	A8: Extraction negative pressure	Type of air leak
1	1.98	6.52	15.10	15.20	15.90	16.12	1.70	20.80	I
2	2.05	5.26	13.58	14.27	14.85	15.56	1.70	20.60	I
3	1.89	6.23	14.88	14.94	15.64	15.76	1.80	20.90	I
4	1.95	6.34	6.95	14.56	14.72	15.24	1.90	20.90	II
5	2.04	7.79	8.68	8.75	15.31	16.05	1.80	21.20	II
6	2.12	5.46	5.65	12.05	12.34	12.79	1.50	19.70	II
7	2.05	6.26	6.33	6.52	14.72	15.29	1.80	20.80	II
8	1.78	9.59	10.81	10.93	11.94	17.25	2.20	21.50	III
9	1.87	5.24	5.26	5.34	5.64	16.52	1.70	19.70	III
10	1.79	5.73	6.18	6.27	9.46	15.26	1.60	19.60	III
11	5.67	24.68	24.89	25.35	25.48	25.64	9.70	20.10	No leakage
12	5.14	17.98	18.12	18.34	18.42	18.56	9.80	19.80	No leakage

We classified the 12 test sample data using single NBC identification and an improved naive Bayes extraction borehole identification model. The results of the analysis are shown in Table 10. Single NBC identification identified 3 boreholes with type I leaks, 5 boreholes with type II leaks, and 2 boreholes with type III leaks, but it could not identify boreholes without leaks. Improved naive Bayesian identification successfully identified 2 boreholes without leaks, 3 boreholes with type I leaks, 4 boreholes with type II leaks, and 3 boreholes with type III leaks.

**Table 10:** Improved naive Bayesian identification results.

Borehole number	Y_1_	Y_2_	Y_3_	Y_4_	Y_5_	Actual type of air leakage	Single NBC identification type	Improved naive Bayesian identification type
1	1.92280	0.85626	0.07374	− 0.38545	0.22717	I	I	I
2	1.60666	0.87215	0.23606	− 0.10815	0.07676	I	I	I
3	1.83057	0.85743	− 0.02364	− 0.63877	0.25861	I	I	I
4	1.52035	0.54786	0.35919	− 0.27686	− 0.29476	II	II	II
5	1.68033	0.22065	0.69849	− 0.11895	0.00684	II	II	II
6	0.69447	0.63193	0.41884	0.54398	− 0.58930	II	II	II
7	1.19167	0.18151	0.76912	0.09367	− 0.21040	II	II	II
8	2.09311	− 0.16483	0.12256	− 0.91361	0.38560	III	II	III
9	0.54409	− 0.43729	0.12125	0.24340	0.05829	III	III	III
10	0.56498	− 0.10154	− 0.07867	− 0.04215	− 0.06021	III	III	III
11	0	0	0	0	0	No leakage	0	No leakage
12	0	0	0	0	0	No leakage	0	No leakage

Table 10 indicates that the single NBC identified the leakage of the No. 8 gas drainage borehole factors, which resulted in errors; this approach could not identify whether the gas drainage borehole was leaking. The recall rate was 75%, and the training time of the identification was 0.0045 s. The identification and analysis recall rate of the improved model was type II, and its real type was type III. This was because the mutual influence between the original eight characteristic Bayesian air leakage identification models of gas extraction boreholes was 98.9%, the identification accuracy improved by 31.8%, and the training time decreased to 0.0020 s, which was an improvement of 55%. To further analyse the error rate, a confusion matrix comparison diagram was drawn^43^. It showed that the improved naive Bayesian gas extraction borehole leakage identification model could fully identify the type of borehole leakage in the Xiashijie coal mine, the identification accuracy was high, and the identification rate was fast.

**Figure 9:**
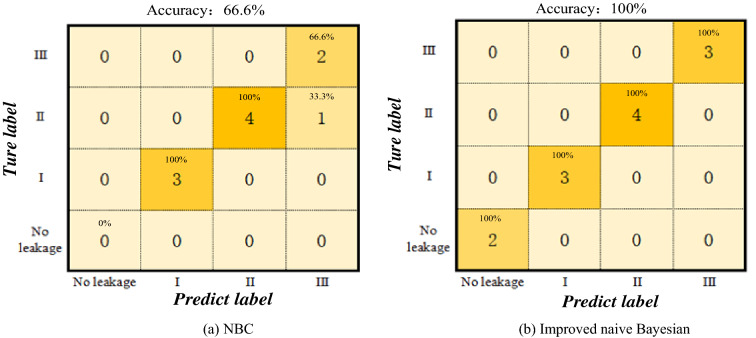
Comparison diagram of the confusion matrix.

As shown in Fig. 9, the single naive Bayesian identification failed to identify boreholes without leaks due to the inability to calculate eigenvalues. This resulted in the effective identification of only 10 out of 12 groups, and type II boreholes were mistakenly identified as type III boreholes. The improved naive Bayes model accurately identified 12 groups of boreholes, and the true value of the improved naive Bayesian model was consistent with the predicted value. Thus, the identification analysis showed that the improved naive Bayesian gas drainage borehole leakage identification model was superior to the single NBC identification analysis. Depending on its superiority, it could more accurately identify the type of air leak and provide further guidance for borehole sealing and repair to improve the efficiency of gas extraction and prevent gas disasters.

## Conclusions

1. Through multisource data fusion theory (MDF) and principal component analysis (PCA), the traditional naive Bayes method was improved, and an enhanced naive Bayes air leakage identification model of gas drainage boreholes was constructed. The new model overcame the shortcomings of the naive Bayes method that could not accommodate missing and nonstandard data and eliminated the misevaluation caused by the superposition of a large amount of feature information in the process of identification of leaks in gas drainage boreholes.
2. The model was applied to the 229 working face of the Xiashijie Coal Mine. Combined with 8 types of characteristic information of gas drainage boreholes. Thirty groups of 240 gas drainage borehole data were divided into training samples and test samples for analysis, and 12 groups of gas drainage borehole test sample data were successfully identified, including 2 boreholes without leaks, 3 boreholes with type I leaks, 4 boreholes with type II leaks, and 3 boreholes with type III leaks, which were consistent with the conditions of the actual gas drainage boreholes. Thus, this study provides a basis for improving gas drainage efficiency and ensuring safe mining in the Xiashijie Coal Mine.
3. The feasibility of the model was verified by the hold-out method. The recall rate of model identification analysis was 98.9%, and the running time was 0.0020 s. Compared with the single naive Bayes method, the operation rate increased by 55%, and the identification accuracy increased by 31.8%. The improved model filled the gap related to the determination and identification of leaks in boreholes and provides a theoretical basis for the evaluation of the quality of sealing and borehole repairs.

## Data Availability

All data generated or analysed during this study are included in this published article.
